# Addressing Tactical Challenges in Embedding Tele-Savvy in a Pilot Pragmatic Trial

**DOI:** 10.1093/geroni/igab046.662

**Published:** 2021-12-17

**Authors:** Kenneth Hepburn, Carolyn Clevenger, Karina Berg, Richard Fortinsky

**Affiliations:** 1 Emory University, Atlanta, Georgia, United States; 2 University of Connecticut, Farmington, Connecticut, United States; 3 University of Connecticut School of Medicine, Farmington, Connecticut, United States

## Abstract

This presentation describes how we plan to address the challenges of testing an evidence-based caregiver program in a real-world setting without the infrastructure and personal contact of a typical RCT. Instead of screening participants for eligibility, clinic staff will pre-identify participants whom clinicians then confirm. Each clinic will include Tele-Savvy as standard of care; we will thus be able to obtain IRB approval for a waiver of consent. By securing agreement from each clinic to incorporate a small set of standard instruments (e.g., Pearlin Caregiver Competence scale) into their standard operating procedure of routinely collecting caregiver data gathering, we will be able to eliminate the need for a central research staff to conduct baseline and follow-up interviews and will, instead, collect key outcome data through the electronic health record. Instead of in person training of Tele-Savvy facilitators, we will rely on an online training program and facilitator manuals.

